# The role of subclinical psychopathic traits on experimentally induced self- and other-compassion

**DOI:** 10.3389/fnbeh.2022.948129

**Published:** 2022-11-08

**Authors:** Jill Lobbestael, Vanessa L. Freund, Nicole Geschwind, Cor Meesters, Frenk P. M. L. Peeters

**Affiliations:** Department of Clinical Psychological Science, Faculty of Psychology and Neuroscience, Maastricht University, Maastricht, Netherlands

**Keywords:** psychopathic traits, compassion, self-compassion, other-compassion, state compassion

## Abstract

Psychopathic traits come with high levels of anger and aggression. Since previous studies showed that compassion can mitigate both anger and aggression, the current research puts compassion forward as a possible target to alleviate psychopathy’s destructive patterns. Specifically, the present study explored the influence of subclinical psychopathic traits–as well as their three subcomponents egocentricity, callousness, and antisociality–on the efficacy of experimentally induced self-compassion (SC) and other-compassion (OC). This manuscript is part of a larger study in which student and community participants (*N* = 230, *M*_*age*_ = 27.41, 65.2% female) completed a psychopathic trait questionnaire to assess their dimensional level of psychopathy, filled out state SC and OC questionnaires, and were randomized to participate in an experimental self- or other-compassion induction. It was expected that psychopathic traits would positively relate to increases in SC but negatively relate to increases in OC. Baseline levels of both SC and OC negatively related to psychopathy. Overall, as expected, the results on change scores show that subclinical psychopathic traits positively related to a stronger increase in SC, irrespective of the type of compassion induction. This positive relation between a stronger increase in SC and psychopathy total and callousness was more pronounced after the SC induction, rather than after the OC induction. Psychopathic traits did not differentially influence changes in OC. One implication of this study is that high psychopathic and callousness traits predispose to profit extra from targeting SC. Furthermore, psychopathic traits do not hinder increasing compassion for others. These findings suggest that compassion is a promising intervention to improve the wellbeing of people with elevated subclinical psychopathic traits and those around them. Although further research is needed to assess the impact of compassion on anger and aggression specifically, and on clinical psychopathy, the current study suggests that both SC and OC may be useful intervention targets in case of elevated psychopathic traits.

## Introduction

Psychopathy is generally described as a multifaceted personality pattern, encompassing dysfunctions that range across interpersonal (e.g., manipulation and dominance), affective (e.g., callousness and lack of empathy), and behavioral domains (e.g., impulsivity, antisocial tendencies; Hare and Neumann, 2008; Patrick et al., 2009; Neumann et al., 2015). However, it has been under debate whether the latter domain constitutes an essential part of psychopathy, as there is evidence that criminal and antisocial behavior should be seen as outcomes of psychopathic traits (see e.g., Boduszek and Debowska, 2016 for an overview). Abundant neurocognitive abnormalities have been documented in psychopathy research, such as low emotional reactivity, poor emotion recognition, and deficient reversal learning (Blair et al., 2018; Viding and McCrory, 2018). Psychopathic traits do not uniquely express themselves in clinical or forensic subgroups. Rather, they reflect dimensional constructs with levels varying in the general population (Guay et al., 2007).

Evidence links psychopathic traits to excessive anger and aggression. Anger has been designated a key emotion in people with elevated psychopathic traits (Kosson et al., 2016), and chronic expressions of anger have been shown across community and incarcerated adults, and adolescent detainees (Hall et al., 2004; Jackson et al., 2007; Kosson et al., 2020). Out of all personality patterns, psychopathy shows the strongest link with aggressive behavior. Offenders diagnosed with psychopathy commit about twice as many violent offenses compared to low-psychopathic offenders (Hare and Jutai, 1983; Porter et al., 2001). Similarly, psychopathy has been designated as one of the primary risk factors for violent recidivism (Walsh and Walsh, 2006). Psychopathic traits also predispose to more indirect aggression, like relational aggression, emotional abuse, and online trolling (Buckels et al., 2014), especially in females (Nicholls et al., 2005; Gray and Snowden, 2016).

Despite the devastating impact anger and aggression pose on individuals with elevated psychopathic traits as well as on their interpersonal relationships, not much is known about mitigating factors. In the current manuscript, we, therefore, focus on the mental and relational wellbeing concept of compassion and on how subclinical psychopathic traits impact the ability to practice compassion. Due to the diversity of theoretical models and methods employed for compassion, there is a lack of consensus on its definition. While not without contention, there is a broad consensus that compassion entails a benevolent emotional response for a person who is suffering, coupled with the motivation to alleviate this suffering (Strauss et al., 2016; Mascaro et al., 2020). The current manuscript will use one well-recognized model of compassion, i.e., that of Neff (2003). Neff postulates self-compassion (SC) consists of three inter-related facets, i.e., kindness (supporting and encouraging), common humanity (considering everyone goes through negative experiences), and mindfulness (validating experiences in an accepting, non-judgmental way). Pommier et al. (2019) applied Neff’s model to the prosocial concept of other-compassion (OC), with the same tri-partite structure. A systematic review on the quality of compassion measures identified Neff’s Self-Compassion Scale as one of the two strongest methodological measures (Strauss et al., 2016). Research evidenced that compassion triggers neuronal activity in the insula, ventral striatum, and medial orbitofrontal cortex (Beauregard et al., 2009; Singer and Klimecki, 2014). SC and OC have been associated with beneficial effects on mental health, like lower anxiety and burnout (Neff et al., 2007; Dev et al., 2020) and increased prosocial behavior, such as better patient care in health settings (Conversano et al., 2020).

The high level of interpersonal difficulties and exploitative nature of individuals with elevated psychopathic traits makes an impairment in OC imminently intuitive. Remarkably, however, empirical support on the narrow concept of OC is lagging. To date, only one study investigated psychopathy’s link to the ability to feel compassion for others. Specifically, female college students were asked how compassionate they felt for a distraught child with a dying father depicted in a movie fragment (Lee and Gibbons, 2017). Psychopathic traits were a strong negative predictor of state compassion, implying that the child’s sorrow left participants with elevated traits unmoved. Ancillary support for an OC deficit in case of elevated psychopathic traits comes from self-report studies showing a negative link between psychopathy and trait affective empathy (Miller and Eisenberg, 1988; Mullins-Nelson et al., 2006; Lishner et al., 2015)–a partly overlapping concept implying truly experiencing the emotions of another (Brouns et al., 2013). From a neurobiological perspective, there is evidence that individuals with psychopathic tendencies who are hypo-aroused in response to others’ distress also show decreased bodily arousal to their own distress. This suggests the presence of a general hypo-responsive neurocircuitry in psychopathy-prone individuals, buffering them against stress but also preventing them from binding with others (see Shirtcliff et al., 2009 for a review). To our best knowledge, the link between psychopathic traits and SC has not been empirically addressed.

Indirect evidence of compassion’s potential as an antidote for psychopathic behavior comes from research linking increased SC levels to lower both anger and aggression–two imminent markers of psychopathy. Angry rumination and recent anger, for example, were negatively related to self-reported SC (Fresnics and Borders, 2017). Similarly, individuals’ reported SC levels predicted lower anger in response to provocative vignettes (Neff and Vonk, 2009), as well as when recalling upsetting autobiographical situations (Reis et al., 2015). Trait aggression also showed a negative correlation with SC (Fresnics and Borders, 2017), both reactively and proactively motivated (Barry et al., 2015), and in the context of romantic relationships (Neff and Beretvas, 2013). At the behavior level, higher SC levels related to participants sticking fewer needles in a voodoo doll representing a rejecting person (Miyagawa and Taniguchi, 2022). Remarkably, all these studies focus exclusively on SC. On the one hand, this is curious given that conceptually, the interpersonal focus of anger and aggression makes their association with OC even more likely than with SC. On the other hand, the dominant focus of general compassion literature has traditionally been on SC instead of OC. So rather than implying an absent association between OC and anger/aggression, there has simply been a lack of empirical focus on OC. One notable exception is research on the impact of therapeutic compassion workshops that were designed to improve SC and OC in violent criminals. Both self- and partner-reported aggression showed a steeper decrease following this workshop compared to cognitive behavior therapy (Stosny, 1995; Murphy et al., 2005). Furthermore, our group recently experimentally induced either SC or OC in a community sample and showed that both inductions not only increased their corresponding state effect, but also their complimentary effects (e.g., SC induction also led to amplified state OC). This suggests that SC and OC mutually feed on and enforce each other (Freund et al., under review).

Given the potential benefits, the current study will assess the influence of subclinical psychopathic trait levels on induced changes in state SC and OC. A mixed sample will be assigned to SC or OC writing exercises to explore if subclinical psychopathic traits facilitate or hinder the acquisition of SC or OC. By doing so, this study will fill the void in the literature on understanding the link between psychopathic traits and (state) compassion. Given the self-centered focus of psychopathy, these traits were expected to facilitate SC. Because of the high-conflict relationships people with elevated psychopathic traits tend to have, and previous studies evidencing impairments in OC (Lee and Gibbons, 2017) and affective empathy (Lishner et al., 2012), we expected that psychopathic traits will act as a suppressor of OC. Within the OC concept, we differentiated whether OC encompasses a focus on the suffering of all other human beings (thus, in *general*) or on an individual person that is a part of one’s social circle (such as a friend or family member, thus, *specific*). We consider this to be a relevant distinction within OC, given that the egocentric focus of psychopathy might predispose to a particular protection of relevant, close targets and in-group members (Leibowitz, 1997; Mihailides et al., 2017; Cichocka and Cislak, 2020). Hence, psychopathic traits were expected to suppress specific OC (directed toward an individual close to oneself) less than general OC (directed toward the general population). Consequently, we hypothesized that

(i) Subclinical psychopathic traits will facilitate increases in SC following the inductions. This will be more prominent after the SC than after the OC induction.

(ii) Subclinical psychopathic traits will suppress increases in OC following the inductions. This will be more prominent for general OC than specific OC.

## Materials and methods

The data was collected as part of a larger research into the malleability of compassion (see Freund et al., under review). The pre-registration can be found here: https://osf.io/8xdrh/?view_only=eae5b63fa28f400e9b3f65979b53230d. Only materials, procedures, and analyses relevant to the current research questions are described in this manuscript. The dataset and syntax can be found on https://osf.io/6e2vz/?view_only=74acc5572d2a4f958f753eea991613ba.

### Participants

From the original sample of *N* = 273, *N* = 43 were excluded based on failing the attention checks and on not adhering to induction instructions. The final sample consisted of *N* = 230 participants (65.2% female) with a mean age of 27.41 (*SD* = 10.85; range 18–65). About 32.0% of participants were German, 28.7% Dutch, 8.7% Belgian, and 30.4% were of 27 other nationalities. Most (37.4%) participants’ highest completed education was high school, and 37.4% completed university (32.2% Bachelor; 19.1% Master). The sample consisted of 58.3% students, 28.7% were employed, and 13% were otherwise engaged. Most participants were in a relationship or married (51.3%) or single (47.4%). The majority (96.5%) of our sample did not take psychoactive medication. The research was available in Dutch and English, and most participated in English (71.3%).

### Materials

#### Psychopathy

Psychopathic traits were assessed with the Levenson Self-Report Psychopathy Scale (LSRP). The LSRP is a preferable psychopathy measure in general population samples, given that its focus does not lie on rather pathological, explicit, and overt antisocial behavior (Garofalo et al., 2019). The LSRP is a preferable psychopathy measure in general population samples, given that its focus does not lie on rather pathological, explicit, and overt antisocial behavior (Garofalo et al., 2019). Although originally containing 26 items (Levenson et al., 1995), we used the 19- items version that has been shown to be superior in reflecting the LSRP three-factor model of psychopathy, i.e., egocentricity, callousness, and antisociality (Garofalo et al., 2019; Wissenburg et al., 2020). The 19 items were scored on a 4-point Likert scale ranging from 1 (completely disagree) to 4 (completely agree). In a general and high-risk community sample, internal consistency of the total score was found to be between α = 0.79 and 0.85, and between α = 0.52 and 0.86 for its subfactors (Garofalo et al., 2019; Wissenburg et al., 2020).

#### State self-compassion

The state Self-Compassion Scale (Neff et al., 2021) was used, in which participants were asked to think of a painful or difficult situation in their life while answering the questions. The specific instruction was: *“Think about a situation you are experiencing right now that is painful or difficult. It could be some challenge in your life, or perhaps you are feeling inadequate in some way. Please indicate how well each statement applies to how you are feeling toward yourself right now as you think about this situation.”* The scale consisted of 18 items scored on a 5-point Likert scale ranging from 1 (not at all true for me) to 5 (very true for me). Note that for all state compassion scales, we used the total scores (i.e., summing the positive and negative compassion items, see e.g., Phillips, 2021). In a student and community sample, internal consistency of the total score was found to be between α = 0.88 and 0.94, and its composite reliability between *CR* = 0.93 and 0.97 (Neff et al., 2021).

#### State other-compassion (general)

The current authors developed the General State Other-Compassion scale by adapting the Compassion Scale items (Pommier et al., 2019) to match the language in the state Self-Compassion Scale (Neff et al., 2021). The questionnaire asked participants to indicate how they feel about people in general (everyone, strangers, neighbors, etc.) in the current moment. The specific instruction was: *“Please think about a situation someone you know is experiencing right now that is painful or difficult. It could be some challenge in their life, or perhaps they are feeling inadequate in some way. Please indicate how well each statement applies to how you are feeling toward other people in general right now.”* The final version included 16 items, scored on a 5-point Likert scale ranging from 1 (not at all) to 5 (very true for me). In the current sample, internal consistencies were acceptable, with α = 0.70 for the pre-measure and α = 0.74 for the post-measure.

#### State other-compassion (specific)

The current authors generated the State Specific Other-Compassion scale by adapting the Compassion Scale items (Pommier et al., 2019) to match the language in the state Self-Compassion Scale while consulting with Kristin Neff, who developed previous (self)compassion scales. The instructions asked participants to think about a difficult or painful situation someone they know (e.g., family member and friend) is experiencing while answering the items. The specific instruction was: *“Please think about a situation that someone you know is experiencing right now that is painful or difficult. It could be some challenge in their life, or perhaps they are feeling inadequate in some way. Please indicate how well each statement applies to how you are feeling toward that specific person right now as you think about their situation.”* It included 16 items, which were scored on a 5-point Likert-scale ranging from 1 (not at all) to 5 (very true for me). In the current sample, internal consistencies were acceptable, with α = 0.70 for both the pre- and post-measures.

#### Self-compassion induction

The Self-Compassionate Mindset Induction (Neff et al., 2021) was used. Participants were asked to think of a specific situation that was currently painful or difficult for them. The induction was composed of the three positive components of SC, i.e., mindfulness, common humanity, and kindness. Each of the three writing prompts encouraged participants to write at least 200 words. Participants were instructed to take a few slow, deep breaths, as they were reading what they had written. The writing task was followed by an attention check in which participants were asked to indicate what was asked of them during the induction, i.e., to write about their feelings in an accepting and validating fashion (i.e., mindfulness), to consider that going through a difficult time is part of being human (i.e., common humanity), and to write words of encouragement to themselves or a friend (i.e., kindness).

#### Other-compassion induction

Other-compassion was facilitated by developing the Other-Compassionate Mindstate induction, which the current authors adapted to mirror Neff et al.’s (2021) SC Mindstate induction in consultation with Kristin Neff. The wording of the SC induction was changed to facilitate OC by instructing the participants to think of a situation that was currently painful or difficult for someone they know while asking them to relate to this person’s hardship. The induction followed the three positive components of OC: (1) writing about what thoughts and emotions might come up as they think about the other person’s difficult situation (mindfulness), (2) writing about how the other’s difficult situation is part of being human (common humanity), and (3) writing feelings of care, kindness, and understanding to the other person (kindness). Each writing prompt instructed participants to write at least 200 words addressed to their chosen person; 52.2% chose a friend, 13.5% a parent, and 7.4% a grandparent. The rest chose either siblings, distant relatives, or colleagues. Participants were instructed to take a few slow, deep breaths, as they were reading what they had written. The last step included an attention check similar to the one in the SC induction.

### Procedure

Participants were recruited at Maastricht University and on Facebook through flyer advertisement, and *via* snowball sampling, and invited to individual 1-h timeslots. After the first 10 participants, recruitment and testing moved online due to COVID-19 restrictions. The study was announced to focus on “*How to deal with situations*”, and it was explained that the goal of this study is *“to understand the different ways people think about and deal with difficult situations. These can be situations they deal with themselves, or others they know. Further, we want to find out how certain personality traits might affect the way people process difficult situations.”* Participants were stratified based on their sex and student/general population status to ensure an even distribution between these two inductions. Participants were informed about the study procedures and gave written consent. Participants then completed the demographical data and the LSRP. Participants were randomly assigned to either the SC induction (*N* = 115) or the OC induction (*N* = 115). Next, participants filled out attention checks, the pre-induction state measures, and the induction. Once the writing task was complete, participants were asked to breathe deeply and reflect on their writing and completed an addition attention check, which took about 5 min. Finally, they completed the post-induction state measures (i.e., state self-compassion; state general other-compassion; and state specific other-compassion) and a manipulation check. The order of the state measures differed depending on the induction allocation (for SC: pre-state general OC, pre-state specific OC, pre-state SC, induction, post-state SC, post-state general OC, and post-state specific OC; for OC: pre-state SC, pre-state general OC, pre-state specific OC, induction, post-state specific OC, post-state general OC, and post-state SC). Upon completion, participants were debriefed and received either university participation credits or a €12.50 voucher. The Ethics Review Committee Psychology and Neuroscience (ERCPN) of Maastricht University approved the research (218_11_02_2020).

### Data preparation

Participants were included in the final sample if they passed the attention check, were oblivious to the research aim, and understood the induction writing task. Two independent raters assessed the writing task for adherence to the instructions. Out of the initial 273 participants, 33 were excluded because of poor adherence. The inter-rater reliability of the two independent raters (2,k ICC for absolute agreement) was excellent, *r* = 0.84 (Cicchetti, 1994). Additionally, 10 participants were excluded due to failing the attention checks (final sample *N* = 230).

### Statistical analyses

Based on a G*power calculation for linear multiple regression, fixed models, *R*^2^ increase with a rather small effect size of *f*^2^ = 0.06, power = 0.95, and alpha = 0.05 with a total number of three predictors, we required a sample size of at least *n* = 219 participants.

General and specific OC were averaged to calculate a total OC score. To assess internal reliability of the subscales, McDonald’s omegas (ML) were calculated using the Statistical Package for the Social Sciences (SPSS) extension ‘Omega, Alphas, and All Subset Reliability Procedure’s Version 1.0 by Hayes and Coutts (2020). To test for baseline differences between the average psychopathy scores of both induction groups, independent samples *t*-tests were completed. Pre-to-post induction change scores for each state measure were calculated (post- minus pre-measure). Overall, the higher the change score, the stronger the increase in compassion.

To test the main hypotheses, the SPSS extension PROCESS macro by Hayes (v.2.5.3; Hayes, 2017) was used, where change scores in compassion were used as *Y* variables, the induction group as *X* variable, and the psychopathic traits (total and subscales) as moderator variables *W*. This resulted in 16 regression moderation analyses: four analyses (one for the total psychopathic trait score, and three for the additional subscales) for each of the four different change scores (SC, total OC, general OC, and specific OC). Model number 1 was applied, with heteroscedasticity-consistent inference (HC3), centering for continuous variables, the Johnson–Neyman method, and *R*^2^s were calculated for effect sizes. Probing within the PROCESS macro analyses split the psychopathic traits into low, medium, and high scores based on ±1 standard deviations.

To lower Type I error chances, we adjusted the critical *p*-value using Benjamini and Yekutieli’s False correction (B–Y method, Narum, 2006). For four regressions per compassion concept (psychopathy total and its three subscales), this adjusted alpha level was 0.024.

## Results

Psychopathy, and state and change compassion scores are presented in Table 1, for the total sample and per induction condition; as well as the internal reliability levels of all scales. Independent samples *t*-tests were conducted to explore subclinical psychopathic trait differences and pre-induction state compassion levels between the conditions. No significant baseline differences were detected within the psychopathy trait or pre-induction scores. Based on face validity, the mean psychopathic traits scores seem comparable to those of other research with similar sample characteristics (Christian and Sellbom, 2016; Maheux-Caron et al., 2020). The internal reliability values of the callousness and antisocial LSRP subscales were rather low, which might be due to the small number of scale items (e.g., Taber, 2018). Still, these levels are comparable to that of other studies (Wissenburg et al., 2020), and still exceeding the generally accepted level of >0.50 (Watkins, 2017). Inter-correlations between the study variables are shown in Table 2. Psychopathy total as well the subscales showed a significant negative correlation with baseline levels of SC, and general and specific OC. The only exception was the non-significant link between baseline specific OC and the egocentricity psychopathy subscale.

**TABLE 1:** Descriptives of variable averages (Mean and SD) and independent samples *T*-tests.

	Mean (Std. Deviation)			Reliability	*T*-Test	
	Total *N* = 230	SC induction *N* = 115	OC induction *N* = 115	Mc Donald’s omega	*t*	*p*
**Psychopathic traits**						
Total	1.99 (0.46)	1.94 (0.44)	2.04 (0.48)	0.79	–1.55	0.56
Egocentricity	1.88 (0.55)	1.84 (0.54)	1.91 (0.57)	0.75	–1.00	0.95
Callousness	1.77 (0.58)	1.74 (0.58)	1.79 (0.58)	0.50	–0.57	0.49
Antisociality	2.40 (0.66)	2.31 (0.63)	2.48 (0.68)	0.51	–2.05	0.66
**Pre-induction**						
State self-compassion	3.51 (0.74)	3.42 (0.73)	3.59 (0.74)	0.91	–1.72	0.96
**State other-compassion**						
Total	4.15 (0.49)	4.18 (0.50)	4.13 (0.47)	*	0.81	0.52
General	4.03 (0.58)	4.09 (0.58)	3.96 (0.58)	0.86	1.73	0.61
Specific	4.28 (0.53)	4.26 (0.54)	4.29 (0.52)	0.84	–0.40	0.97
**Post-induction**						
State self-compassion	3.81 (0.64)	3.89 (0.59)	3.73 (0.68)	0.90		
**State other-compassion**						
Total	4.28 (0.50)	4.26 (0.52)	4.31 (0.49)	*		
General	4.16 (0.62)	4.14 (0.62)	4.17 (0.61)	0.91		
Specific	4.41 (0.50)	4.37 (0.51)	4.44 (0.49)	0.85		
**Change**						
Self-compassion	0.30 (0.52)	0.46 (0.56)	0.14 (0.43)	*		
**Other-compassion**						
Total	0.13 (0.32)	0.08 (0.30)	0.18 (0.33)	*		
General	0.13 (0.45)	0.05 (0.41)	0.21 (0.48)	*		
Specific	0.13 (0.32)	0.11 (0.32)	0.15 (0.32)	*		

Psychopathic traits and pre- and post-induction states had possible ranges of 1–5.

*These scales represent calculated variables that do not have their own scale items; thus, reliabilities cannot be calculated.

**TABLE 2:** Pearson correlations between psychopathic traits and pre-induction compassion states.

Pre-induction states	Psychopathy Global	Psychopathy egocentricity	Psychopathy callousness	Psychopathy antisociality
Self-compassion	−0.36**	−0.29**	−0.16*	−0.35**
**Other-compassion**				
Total	−0.34**	−0.25**	−0.34**	−0.24**
General	−0.40**	−0.33**	−0.36**	−0.27**
Specific	−0.18*	–0.11	−0.23*	−0.15*

*p < 0.05, **p < 0.001.

The main effects of the type of induction can be seen in the induction column of Table 3 and Supplementary Table 1. The main induction effect was significant for SC, total OC, and general OC, but not for specific OC. A significant induction effect means that one induction was significantly better at improving the compassion state than the other. Specifically, the results show that the SC induction was significantly more successful at improving SC than the OC induction, as evidenced by the negative value of the main induction effect (given that the SC induction was coded as 1, and OC induction as 2). Additionally, the OC induction led to significantly greater improvements in total and general OC compared to the SC induction, as evidenced by the positive value of the main induction effect. For further background on the inductions’ ability to malleable compassion, please see Freund et al. (under review).

**TABLE 3:** Significant moderation regression analyses for self-compassion change, induction, and psychopathic traits.

Change	Model	Induction			Psychopathy			Interaction: induction x psychopathy				Effect size
State	#	*B*	*t*	*p*	*B*	*t*	*p*	Score	*B*	*t*	*p*	*R* ^2^
Self-compassion	1				Total							
		−0.34	−5.10*	<0.0001	0.66	2.57*	0.01		−0.34	−2.40*	0.02	0.13
								Low	−0.18	−2.07	0.04	
								High	−0.50	−4.87*	<0.0001	
	2				Egocentrism							
		−0.33	−4.95*	<0.0001	0.35	1.48	0.14		−0.18	−1.34	0.18	0.11
	3				Antisociality							
		−0.34	−5.23*	<0.0001	0.41	2.08	0.04		−0.20	−1.80	0.07	0.13
	4				Callousness							
		−0.33	−4.95*	<0.0001	0.48	2.31*	0.02		−0.28	−2.30*	0.02	0.13
								Low	−0.17	−1.94	0.05	
								High	−0.49	−4.60*	<0.0001	

*Significant at False Discovery Rate (FDR): *p* = 0.0240.

**FIGURE 1:**
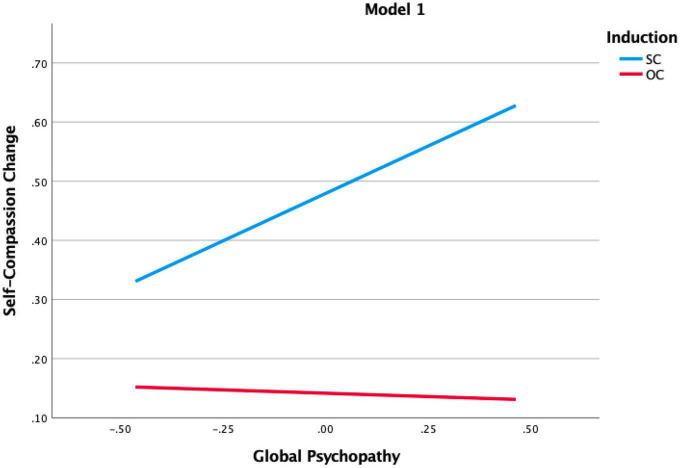
Probing of significant interaction in model 1.

**FIGURE 2:**
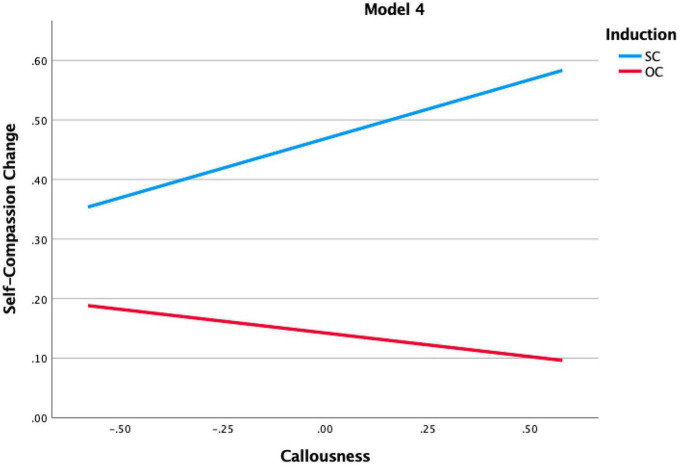
Probing of significant interaction in model 4.

The hypothesis that psychopathic traits would positively relate to an increase in SC was supported. Table 3 shows a main effect of total psychopathic traits, as well as of the subfactor callousness. This implies that the higher these traits, the higher the increase in SC, irrespective of whether the induction was aimed at increasing SC or OC. Furthermore, the interaction effects between the inductions and psychopathic traits were significant for both total psychopathic traits and callousness traits. Probing as a *post-hoc* analysis of these interaction effects (see Figures 1, 2) showed that the higher total psychopathic traits, as well as higher callousness traits, led to the largest increases in SC after the SC induction.

The hypothesis that psychopathy would relate to an inferior increase in OC was not supported, as none of the main trait nor interaction effects OC change scores were significant (see Supplementary Table 1). This implies that psychopathic traits neither facilitated nor suppressed OC change after the inductions.

## Discussion

The present research tested whether psychopathic traits facilitate compassion for the self and hinder compassion for others. It investigated the efficacy of SC and OC inductions at increasing SC and OC state levels, while taking trait levels of subclinical psychopathy into account. By doing so, this research fills the void of understanding how psychopathy relates to the malleability of SC and OC. This is relevant as psychopathic traits can be devastating to both interpersonal relationships and the self. Compassion could be a wellbeing related antidote that can alleviate the strain put on the self and others posed by individuals with psychopathy traits.

Correlations showed that all psychopathic trait subscales were negatively related to all compassion states at pre-induction. This follows previous research that found that psychopathic traits related to reduced general OC (Lee and Gibbons, 2017) and broadens this relation by further linking specific OC deficits to psychopathic traits. Furthermore, considering that most psychopathological traits, and concepts such as increased egocentrism and social disconnectedness, which are highly related to psychopathy, are linked to reduced SC (Neff and McGeehee, 2010; Athanasakou et al., 2020), the negative correlations between psychopathic traits and SC follow expectations.

The main finding is that subclinical psychopathic traits generally facilitate SC after practicing compassion, corroborating our first hypothesis. Specifically, participants were asked to remember a painful situation encountered in the past that either they, or someone they know, experienced. They were stimulated to accept uncomfortable feelings, understand how that is part of being human, and to express support and non-judgmental understanding. Our findings show that engaging in compassion in this way led to steeper increases in state SC in case of elevated psychopathic traits. The increase was even stronger following a compassion intervention that focused on one’s own hardship. This interaction effect appeared to be particularly driven by callousness, i.e., psychopathy’s immoral component of not being bothered by lying, cheating, and hurting others to achieve one’s goal. Results suggest that a psychopathy-related self-permissive moral attitude also predisposes to high self-care and -kindness, following a compassionate reflection on autobiographic past struggles.

Psychopathic traits did not differentially influence the impact of the OC induction. This disconfirmed our second hypothesis of subclinical psychopathic traits hindering OC following induced compassion. The pattern also appeared unaffected by whether participants were asked to direct their compassion for other people in general or those of a specific loved one. At first sight, this finding contradicts those of Lee and Gibbons (2017), who found psychopathic traits to relate to less compassion felt for a boy in distress observed in a movie fragment. One clear difference though with the current study is that we did not merely assess OC but instructed participants to *engage* in OC. Collectively -conditional on the replication of these results- both findings can indicate that individuals with elevated psychopathic traits are not naturally inclined to exert OC, but, when actively encouraged to do so, are, in fact, very capable of experiencing OC.

Overall, psychopathic traits were negatively related to baseline levels of SC and OC. This implies that although subclinical psychopathic traits relate to lower compassion in general, these traits also facilitate increases in SC and do not impair increases in OC when instructed to practice compassion. In other words, psychopathic traits could predispose to a compassion deficit but, simultaneously, also leave room for increasing compassion when directly targeted. Notably, the egocentricity scale of psychopathy was unrelated to baseline specific OC, which might imply that higher levels of this subscale may safeguard against impaired compassion for close others.

The fact that our study findings do not position a positive concept like compassion as polar opposites to psychopathy is not new. Certain psychopathy aspects have, for example, also shown positive associations with happiness or personal growth (Durand, 2018). This implies that it is crucial for research to investigate the context in which compassion is expressed and that there may be conditions under which individuals who are generally lacking compassion, such as those with elevated subclinical psychopathic traits, may transition from lacking compassion for others to becoming more compassionate.

One important clinical implication is that compassionate behavior could be trained when targeted directly, even in individuals with increased subclinical psychopathic traits who are believed to be resistant to healthy intra- and interpersonal wellbeing. The fact that psychopathic traits do not hinder OC designates compassion as a potential therapeutic focus for improving interpersonal relationships. Our findings suggest that, in case of elevated non-clinical psychopathic traits, relational wellbeing would profit from both self- and other-focused compassion interventions, while personal wellbeing would primarily benefit from promoting SC. It has to be noted that there is also a chance that promoting SC will exert a disadvantageous effect on people with clinical levels of psychopathy. Although we previously found (Freund et al., under review) that SC leads to more OC, we do not yet know whether such a mutually enforcing effect also occurs within people who score higher on psychopathic traits. In fact, it might be that the presence of psychopathic traits re-shape the SC-OC link from a linear into a curved linear pattern. While SC is essentially unselfish and balanced, there may be a tipping point at which high SC is misinterpreted and might further enhance egocentricity and therefore install an excuse to misbehave toward others in case of clinical levels of psychopathy.

Relatedly, it is important to stress that in individuals with clinical levels of psychopathy, increasing compassion for others in the longer term will be highly challenging. The current study does not speak on this as we used a single, short-lived compassion induction. A lasting compassion increase requires a shift in the basic social orientation of those with increased psychopathic traits, from power (i.e., getting ahead) to caring (i.e., getting along). The latter relies on a fundamentally different neurological system, like the limbic circuitry, modulated by hormones such as oxytocin (Shirtcliff et al., 2009). Such a motivational change is highly challenging and can only be reached by intensive cognitive therapies (Mascaro et al., 2017), like Compassion Focused Therapy (CFT, Gilbert, 2020; Gilbert and Simos, 2022). CFT was developed for people with complex mental problems and antisocial behavior as an evolution-informed biopsychosocial model. Here, psychopathic traits are conceptualized as an evolutionary response to dealing with hard rearing, as the human brain is designed to survive (Ribeiro da Silva et al., 2020, 2021b). Further, CFT is based on the notion that compassion depends on the committed motivation to orient to others’ suffering and alleviate it (Poulin, 2017). Psychopaths often display fear of compassion (see Kirby et al., 2019) due to unprocessed trauma (see e.g., Ribeiro da Silva et al., 2020). The compassion for patients’ traumatic past conveyed *via* the therapeutic relationship in CFT forms a pre-requisite for patients to treat others compassionately (Ribeiro da Silva et al., 2020), conceptualizing compassion as a reciprocal flow (Gilbert, 2014). This aligns with evidence that children with callous traits can be guided away from more psychopathic futures with affection, love, and care (Henry et al., 2018). The effectiveness of CFT has been shown in juvenile detainees (Gilbert, 2017; Ribeiro da Silva et al., 2021a).

Previous, albeit cross-sectional, research implies that SC can act as an antidote for anger and aggression (e.g., Neff and Vonk, 2009; Reis et al., 2015; Fresnics and Borders, 2017). This makes sense for several reasons. First, exerting compassion implies reminding people of their connectedness to others, which has been shown to buffer aggression even toward people that reject you (Twenge et al., 2007). Second, adopting compassion in relationships is postulated to soothe one’s emotional state (Neff, 2011), framing compassion as an emotion regulation strategy (Neff, 2011). Aggression research indeed pinpoints emotion regulation as a key preventive aggression factor (Roberton et al., 2012). Finally, research suggests that SC promotes acceptance of a partners’ weakness (Zhang et al., 2020) and helps people find a compromise between their own and others’ needs in interpersonal conflicts (Yarnell and Neff, 2013). Whether compassion is a viable target to reduce anger and aggression in individuals with elevated psychopathic traits is still an open question. To date, one study had the comparative aim to assess the protective effect of SC on the link between narcissism–sharing a self-centered preoccupation with psychopathy- and aggression. Cross-sectional moderation analyses in youngsters failed to support such a protective SC function, though the sample was small and male only (Barry et al., 2015). Importantly also, SC was assessed at the trait level here, omitting investigation of how SC augmentation impacted aggression. Thus, while currently unexplored territory, we tentatively put compassion focused interventions forward as promising targets for reducing psychopathy-related anger and aggression (cf. Fresnics and Borders, 2017).

Strengths of the current study include that both SC and OC were induced and measured according to the empirically supported three-component structure of compassion. Further, the current research incorporated the distinction between *general* and *specific* OC, which allowed us to assess whether increases in OC were specific to close others or generalized to others in general. Additionally, the inclusion of psychopathic subscales provided for fine-grained analyses into its multifaceted nature. One limitation of our study is that the OC state measures have not been validated previously. The results indicate that they are able to measure state OC well, but future research is needed to confirm this. Moreover, this experiment’s cross-sectional nature prevents assessing the longevity of improvements after the inductions. To further validate these findings, future research should replicate the methods in a larger sample and in individuals with pathological levels of psychopathy to determine whether findings are generalizable to clinical samples. Future research would also benefit from assessing how fear of compassion relates to psychopathy (Kirby et al., 2019), as well as whether integrating body training practices (which are also part of CFT, see Gilbert, 2020) would further enhance compassion in this sample. Future studies should further investigate the benefits of practising these exercises for prolonged periods and research if compassion changes remain at follow-ups. It would also be advisable to explicitly assess motivation for compassion, as stimulating genuine engagement can be especially challenging for those with increased psychopathic traits. Finally, the research field into compassion is rapidly evolving, with many controversies regarding its definition and assessment (Strauss et al., 2016; Mascaro et al., 2020). In light of this, it should be kept in mind that the current manuscript solely focuses on one–though a well-recognized and internationally used–model out of many available on compassion.

Taken together, subclinical psychopathic traits were shown to facilitate SC, while not hindering OC, following a compassion exercise. One implication of study is that compassion could be a promising intervention for the wellbeing of those with elevated psychopathic and callous traits, as well as for those around them. Given the success of the OC induction for individuals with elevated psychopathic traits, it seems that a lack of compassion for others could be counteracted when OC is directly targeted. While further studies are needed to assess the longer-term effects as well as the impact on psychopathy’s problematic level of anger and aggression, the current study identified compassion as a valid candidate for mitigating psychopathic traits.

## Data availability statement

The datasets presented in this study can be found in online repositories. The names of the repository/repositories and accession number(s) can be found below: https://osf.io/6e2vz/?view_only=4924b854096f43c7a5792520713cfec0.

## Ethics statement

The studies involving human participants were reviewed and approved by Ethics Review Committee Psychology and Neuroscience (ERCPN) of Maastricht University Psychology and Neuroscience (218_11_02_2020). The participants provided their written informed consent to participate in this study.

## Author contributions

VF collected the data, organized the database, and wrote sections of the manuscript. VF and JL performed the statistical analysis. JL wrote the first draft of the manuscript. All authors contributed to the conception and design of the study, manuscript revision, read, and approved the submitted version.
