# Supplementary material for: The role of subclinical psychopathic traits on experimentally induced self- and other-compassion
Source: Front Behav Neurosci. 2022 Nov 8;16:948129. doi: 10.3389/fnbeh.2022.948129 (PMC9681498; doi:10.3389/fnbeh.2022.948129)
Supplement: Supplementary file 1 [file Table_1.docx]

**Table S1.** *Moderation Regression Analyses for Other-Compassion Change (OC), Induction, and Psychopathic Traits.*

| Change | Model | Induction | | | Psychopathy | | | Interaction: Induction x Psychopathy | | | Effect size |
| --- | --- | --- | --- | --- | --- | --- | --- | --- | --- | --- | --- |
| State | # | *B* | *t* | *p* | *B* | *t* | *p* | *B* | *t* | *p* | *R^2^* |
| General  OC | 5 |  |  |  | Global | | |  | | |  |
|  |  | .17 | 2.91* | .004 | -.17 | -.85 | .39 | .04 | .27 | .79 | .04 |
|  | 6 |  |  |  | Egocentrism | | |  | | |  |
|  |  | .17 | 2.89* | .004 | -.10 | -.58 | .56 | -.01 | -.10 | .92 | .05 |
|  | 7 |  |  |  | Antisociality | | |  |  |  |  |
|  |  | .16 | 2.72* | .007 | -.09 | -.67 | .51 | .04 | .48 | .63 | .03 |
|  | 8 |  |  |  | Callousness | | |  |  |  |  |
|  |  | .16 | 2.70* | .008 | -.11 | -.69 | .49 | .06 | .48 | .64 | .03 |
| Specific  OC | 9 |  |  |  | Global | | |  | | |  |
|  |  | .05 | 1.13 | .26 | -.02 | -.16 | .88 | -.003 | -.04 | .97 | .01 |
|  | 10 |  |  |  | Egocentrism | | |  | | |  |
|  |  | .05 | 1.14 | .26 | -.10 | -.83 | .41 | .04 | .61 | .55 | .01 |
|  | 11 |  |  |  | Antisociality | | |  |  |  |  |
|  |  | .05 | 1.04 | .30 | .09 | .84 | .40 | -.06 | -.84 | .40 | .01 |
|  | 12 |  |  |  | Callousness | | |  |  |  |  |
|  |  | .05 | 1.10 | .27 | .02 | .24 | .81 | -.03 | -.43 | .67 | .01 |
| Total  OC | 13 |  |  |  | Global | | |  | | |  |
|  |  | .11 | 2.61* | .01 | -.10 | -.73 | .47 | .02 | .20 | .85 | .04 |
|  | 14 |  |  |  | Egocentrism | | |  | | |  |
|  |  | .11 | 2.61* | .01 | -.10 | -.88 | .38 | .02 | .18 | .86 | .04 |
|  | 15 |  |  |  | Antisociality | | |  |  |  |  |
|  |  | .10 | 2.42* | .02 | .00004 | .001 | .99 | -.01 | -.09 | .93 | .03 |
|  | 16 |  |  |  | Callousness | | |  |  |  |  |
|  |  | .10 | 2.46* | .02 | -.04 | -.41 | .68 | .02 | .20 | .84 | .03 |

*Significant at False Discovery Rate (FDR): *p* = .0240.
